# Clinical Significance of the Prognostic Nutritional Index in Predicting Delirium among Critically Ill Patients: A Retrospective Cohort Study

**DOI:** 10.1155/2024/3807532

**Published:** 2024-05-11

**Authors:** Feifei Xu, Xi Zhong

**Affiliations:** Department of Intensive Care Medicine, West China Hospital, Sichuan University, Chengdu 610041, Sichuan, China

## Abstract

Delirium is a serious and complex problem facing critically ill patients. This retrospective study aimed to explore the association between prognostic nutrition index (PNI) and delirium in critically ill patients in the intensive care unit (ICU). This study was based on the Medical Information Mart Intensive Care IV (MIMIC IV) database. Patients over 18 years of age were enrolled. Univariate and multivariate logistic regression analyses were performed to explore the association between PNI and delirium. Tendency analysis, subgroups analysis, and restricted cubic spline (RCS) were selected to further certify the association. The receiver operating characteristic curve (ROC) was adopted to assess the predictability of PNI to delirium. Propensity score matching (PSM) was used to reduce the interference of confounders. A total of 3,105 patients participated in this study. As the grade of malnutrition increases, the incidence of delirium increases in all models. The odds ratios (OR) of delirium in the fully adjusted model were 1.00 (reference), 1.04 (0.80, 1.36), 1.53 (1.17, 1.99), and 1.93 (1.44, 2.59). Strong U-shaped curves were found with RCS analysis between delirium and PNI in the subgroups of patients over 60 years of age and patients with chronic obstructive pulmonary disease (COPD). After PSM, the ORs of delirium were 1.44 (1.16, 1.79) and 1.53 (1.22, 1.93), respectively, in the univariate and multivariate logistic regression models. PNI is negatively associated with the prevalence of delirium in critically ill adults in the ICU. PNI is an independent risk factor for the incidence of delirium in adults in the ICU.

## 1. Introduction

Delirium is a very common manifestation of brain dysfunction in critically ill patients [1]. It is defined as a disturbance in attention that develops over a short period of time that cannot be explained by another preexisting neurocognitive problems [2–4]. In particular, studies have shown that ICU delirium is associated with increased mortality, prolonged hospitalization and mechanical ventilation, increased costs, and the appearance of cognitive disorders after discharge from ICU [5–8]. Although the disadvantages of delirium have been realized, its prevalence remains high. Based on epidemiological studies, the incidence of postoperative delirium is approximately 45–50%. In addition, it even reaches 80% of patients undergoing mechanical ventilation [9–11]. Therefore, it is of great importance to explore related clinical risk factors for ICU delirium.

To date, extensive research has shown that delirium is connected to multiple stimulating factors. Studies by Marcantonio showed that age was a major demography-related risk factor for delirium [12]. Previous research also highlighted the presence of COPD as a significant risk factor in the development of delirium [13], and the underlying mechanism may be related to chronic hypoxia that promotes brain dysfunction and cognitive decline [14, 15]. Furthermore, trauma was suggested to serve as a predisposition for delirium [16]. Recent evidence recognized that nutrition played a critical role in the development of delirium. For example, a cohort study confirmed that patients overtly malnourished nearly tripled the risk of postoperative delirium [17]. Despite the controversy, a different opinion was that malnutrition was not correlated with delirium onset in older patients after noncardiac surgery [18]. Therefore, the present conclusions about the effect of malnutrition on delirium are inconsistent. Thus, this study was designed to investigate the association between the grade of malnutrition reflected by PNI and the incidence of delirium in patients in the ICU.

PNI, an indicator that includes lymphocyte count and serum albumin, was initially used to evaluate the perioperative immune nutritional status of patients undergoing gastrointestinal surgery [19]. Afterward, PNI gradually became a new predictor of the prognosis of many diseases. For example, low PNI contributes to high adverse events in preeclampsia [20]; it also can predict early mortality in diffuse large B-cell lymphoma [21]. Recent research even documented that PNI can be used to guide the clinician in predicting the 30-day mortality of patients diagnosed with delirium [22]. However, PNI has rarely been studied in association with delirium in the critically ill population admitted to the ICU.

This paper clearly demonstrated that PNI was negatively associated with the incidence rate of delirium in ICU patients. It not only provided the basis of PNI to predict delirium in patients with serious conditions but also reinforced the opinion that malnutrition was a risk factor for delirium in the target patients.

## 2. Methods

### 2.1. Data Source and Study Population

The data source in this article was extracted from MIMIC IV (version 2.0), collecting information about ICU patients at Beth Israel Deaconess Medical Center (BIDMC) between 2008 and 2019. This database mainly contained demographics, diagnosis, clinical and laboratory measurements, treatments, survival data, and other medical data [23].

**Figure 1:**
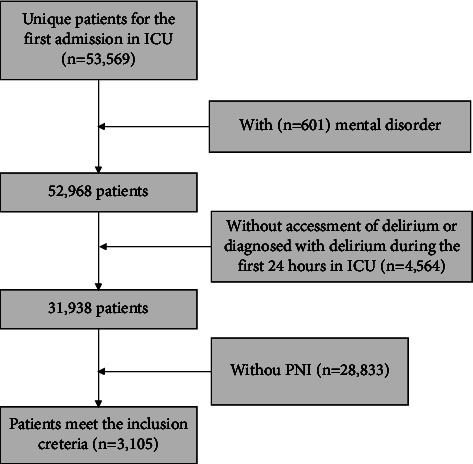
The flowchart of the study population.

Patients older than 18 years were adopted. Furthermore, only the first admission to the ICU was included for analysis. The exclusion criteria were (1) loss of assessment of delirium; (2) without the information of PNI; (3) diagnosed with delirium before or during the first 24 h after ICU admission; and (4) with mental problems (Figure 1). All the courses on how to use the database have been completed by the first author. The consent of an individual patient was waived because the project did not interfere with clinic treatment and protected information was anonymized.

### 2.2. Data Extraction and Data Processing

Medical information was extracted by Navicat Premium (version 16). Medical indexes were collected during the first 24 hours after admission to the ICU. Demographic variables included age, gender, body weight, and height at admission. Basic vital signs referred to heart rate, arterial pressure, respiratory rate, body temperature, and oxygen saturation. Laboratory indexes involved blood glucose, hemoglobin, blood creatinine, calcium, chloride, potassium, prothrombin time (PT), partial thromboplastin time (PTT), aspartate aminotransferase (AST), alanine aminotransferase (ALT), bilirubin, etc. Basic chronic diseases included diabetes mellitus (DM), COPD, and chronic kidney dysfunction (CKD). Delirium was estimated 24 hours after admission to the ICU and established as the primary outcome.

### 2.3. Definition of Delirium and PNI Acquisition

The PNI is an index that shows the nutritional status. It was calculated as PNI = 10 × albumin (g/dL) + 0.005 × lymphocyte count (/mm^3^) [24, 25]. In the MIMIC-IV database, delirium was assessed using the confusion assessment method for the intensive care unit (CAM-ICU) and diagnosed with the Diagnostic and Statistical Manuals of Mental Disorders (DSM-5) [26, 27]. The PNI quartiles were listed as follows: the first quartile: PNI 19.00 and <32.55, marked as grade 4 (G4); the second quartile: PNI < 38.15, marked as grade 3 (G3); the third quartile: PNI < 44.75, marked as grade 2 (G2); and the fourth quartile: PNI 72.60, marked as grade 1 (G1). From G1 to G4, the risk of malnutrition increased in turn.

### 2.4. Statistical Analyses

Data were presented as mean ± standard deviation or median (25–75% quantiles) according to their distribution in continuous variables. Categorical data are shown as proportions (%). One-way analysis of variance, Kruskal–Wallis or chi-square test, was used to test differences between groups. Logistic regression analyses were used to identify the association between PNI and delirium. OR was calculated, and 95% CI of the OR from these models is presented. Model 1 was a univariate analysis; model 2 was adjusted for age, sex, and basic vital signs including heart rate, respiratory rate, systolic blood pressure (SBP), diastolic blood pressure (DBP), body temperature (T), and oxygen saturation (SpO_2_). Model 3 was further adjusted for laboratory findings: serum glucose (Glu), hemoglobin (Hb), serum creatinine (Cre), bicarbonate, calcium, chloride, potassium, PT, PTT, AST, ALT, and bilirubin. Model 4 was further adjusted for basic chronic diseases: DM, CKD, and COPD. Subgroup analyses were performed to further explore the underlying relationship in particular groups. ROC was used to evaluate the performance of PNI for delirium prediction; the cutoff value was calculated and set as the threshold value for grouping in PSM. To reduce the interference of selective bias and confounders, a 1 : 1 PSM analysis was performed using the nearest-neighbor method, with a caliper size of 0.03. The propensity score was calculated using logistic regression analysis. The cut-off value of the standardized mean difference (SMD) was established as less than 0.01, which was considered sufficient balance between the two groups [28]. RCS was used to flexibly model and visualize the nonlinear relationship between PNI and delirium in different groups [29].

All analyses were performed using Stata 16.1 (Stata Corporation, College Station, TX, United States) and the R 4.3.1 software (R Foundation for Statistical Computing, Vienna, Austria). Statistical significance was considered when *P* < 0.05 (two-sided).

## 3. Results

### 3.1. Baseline Characteristics of the Participants

A total of 3,105 patients in the ICU met the inclusion criteria (Figure 1), including 1,780 males and 1,325 females. The median age of the patients in G1 to G4 was 63.20, 68.25, 66.25, and 65.07 years. A total of 1,076 patients underwent delirium and the general incidence was 34.65%. The incidence among the groups was 24.8%, 28.6%, 38.3%, and 46.8%, respectively, showing an increasing trend with the PNI grade. Details are shown in Table 1.

### 3.2. The Association between PNI and Delirium

Four logistic regression models were established. G1 was set as the reference group. Data showed that delirium ORs in all models increased with the growth of nutritional grade reflected by the PNI quartiles, suggesting that critically ill patients with lower PNI had a higher probability of suffering delirium in the ICU. In the univariate model, the risk of delirium in the G4 group increased by 1.45 times compared to the reference group. Similarly, even in the fully adjusted model, there was still a 1.93-fold increase compared to the G1 group (Table 2).

### 3.3. Subgroup Analysis and Interaction Effect Analysis

Subgroup analysis was conducted to testify for the different associations between PNI and delirium in the subgroups. The results showed that the ORs still increased from G1 to G4 in patients who were male, middle-aged (45–60 years), without DM, COPD, or CKD. The trends were not that obvious in the other subgroups. All the *p* values for the interaction were greater than 0.05, indicating that the effect of PNI to the delirium was consistent in these subgroups. The specific results are shown in Table 3.

### 3.4. ROC and Propensity Score-Matching Analysis

**Figure 2:**
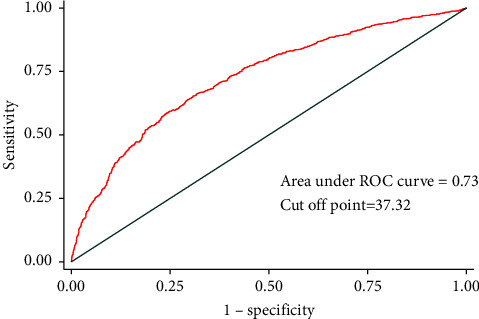
ROC analysis of PNI.

**Figure 3:**
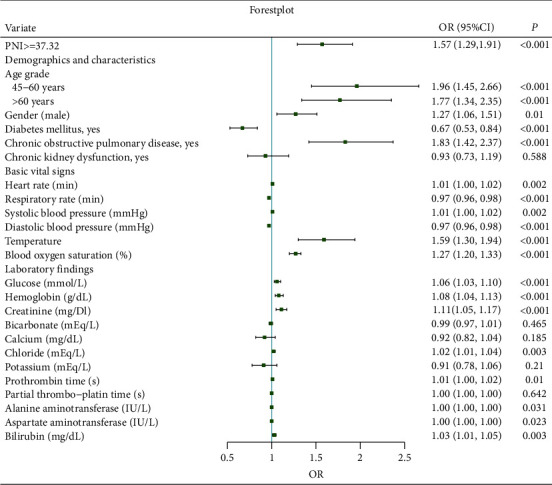
Forest plot for the fully adjusted model. OR: odds ratio.

ROC was utilized to estimate the predictive ability of PNI to delirium.  Figure 2 showed that the AUC was 0.73, and the cut-off value calculated by the Youden method was 37.32. Based on this cut-off point, PNI was divided into two groups: PNI ≥ 37.32 and PNI < 37.32. Logistic regression was performed after the PNI was regrouped. The result showed that the OR was 1.94 (1.67, 2.25), and the *p* value is less than 0.001 in univariate logistic regression. The OR was 1.57 (1.29, 1.91), which is shown in Figure 3.

**Figure 4:**
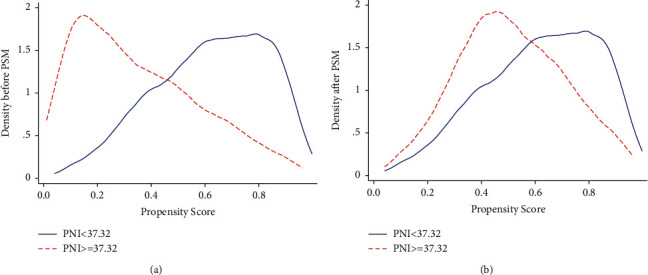
Kernel density plot for PSM: (a) kernel density plot before PSM and (b) kernel density plot after PSM.

To reduce the interference of confounding factors, PSM was performed to further assess the prognostic value of PNI. Propensity scores were calculated using a multivariate logistic regression analysis for baseline differences. Patients with PNI ≥ 37.32 and PNI < 37.32 were matched in a 1 : 1 ratio based on their propensity scores using closest-neighbor matching. In the current research, 0.03 was selected as the caliper width for PSM. Every possible confounder was considered. Kernel density plots of propensity scores were used to show the equivalence between matched patients (Figure 4). The baseline differences in all clinical covariates between the PNI ≥ 37.32 and PNI < 37.32 groups were balanced using a PSM analysis (supplementary figure and table (available here)).

**Figure 5:**
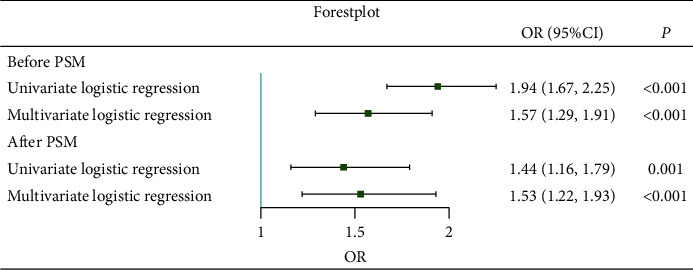
Forest plot of logistic regression after PSM. OR: odds ratio.

Univariate and fully adjusted multivariate logistic regressions were performed. The results showed that the ORs for delirium were 1.44 (1.16, 1.79) and 1.53 (1.22, 1.93), respectively, indicating that the risk of delirium actually increased with the PNI grade even after adjusting the confounders (Figure 5).

### 3.5. Restricted Cubic-Spline Regression

**Figure 6:**
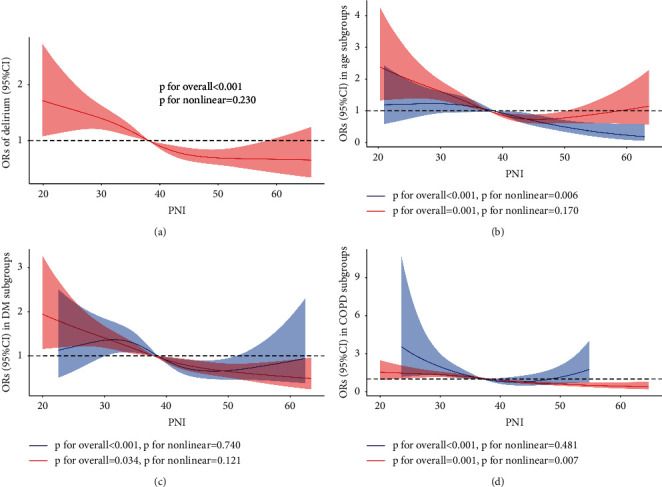
Subgroup analysis of RCS. (a) RCS results for all patients. (b) Subgroup analysis of RCS in age. (c) Subgroup analysis of RCS in DM. (d) Subgroup analysis of RCS in COPD. The RCS regression model was adjusted as model 4. RCS: restricted cubic spline. OR: odds ratio. PNI: prognostic nutritional index. DM: diabetes mellitus; CKD: chronic kidney disease; COPD: chronic obstructive pulmonary disease.

In this section, RCS was used to flexibly model and visualize the relationship between PNI and delirium in different groups. The risk of delirium generally decreased in all participants (Figure 6(a)). The linear relationship with delirium for PNI was suggested (*p*=0.230). To further explore the relationship between delirium and PNI, RCS was performed in the subgroups. Strong U-shaped relationships were found in patients with COPD (*p* for nonlinear = 0.007) and in patients aged over 60 years, indicating that PNI has a dual effect on delirium (Figure 6(b) and 6(d)). No obvious nonlinear relationship was found in the other subgroups.

## 4. Discussion

This study unveiled the negative association between PNI and delirium incidence in ICU patients, suggesting that a low grade of PNI may be a risk factor for delirium in the target population. Meanwhile, in patients aged over 60 or patients with COPD, the affection of PNI to delirium inverted with the growth of PNI . These findings shed light on the influence of malnutritional status on delirium predisposition, which may play a role in the pathological process of delirium.

PNI was an indicator that integrated albumin and lymphocyte count, reflecting both nutritional condition and immune status [30, 31]. Our findings were consistent with other studies indicating negative association between PNI and delirium. For instance, PNI was seen as independent predictors for delirium in aged patients after spinal surgery [32], hip fracture surgery [33], total hip arthroplasty [34], or in noncardiac-surgery patients [35]. In line with these studies, we proved that the risk of delirium was positively associated with a worse malnutrition status classified by PNI. In this paper, all ORs increased from G1 to G4, and tendency analysis showed that the increase had statistical significance. Notably, all *p* values at G2 were also found to be greater than 0.05. There was a possibility that mild malnutritional status does not affect the prevalence of delirium, and this is in accord with the conclusion of Liu [35]. As nutritional risk increased, the accumulated influence of PNI on delirium emerged, and subsequently, all ORs appeared statistically significant at G3 and G4. Furthermore, PSM was conducted to attenuate the affection of confounding factors; and the results of logistic regression after PSM still showed the same trend, improving the robustness of the models. One explanation is that malnutrition is always associated with multifaceted adverse clinical effects. It can carry physiological, sociological, and psychological risk factors at the same time [36–38]. When stressors such as surgery or critical trauma hit, the human body should respond adequately through a variety of physiological mechanisms such as gluconeogenesis or amino acid mobilization to maintain glucose and amino acids in plasma [39–42]. However, without the appropriate physiological reserves to respond, those who are malnourished begin the recovery process at a more vulnerable baseline [38, 43]. For example, the blood glucose supplied to the brain was kept merely at a relatively low level until glycogenolysis started, and this may affect the function of the brain [44, 45]. Another possible reason was that malnutrition may include an impairment of neurotransmission due to thiamine deficiency, which was necessary for normal brain function due to its increased metabolic demand [46]. Moreover, malnutrition is always linked with clinical frailty [47], which had a strong association with postoperative delirium [48, 49]. Given this, as an indicator of nutritional status, PNI is negatively associated with the appearance of delirium. In addition, it should be noted that delirium risk showed a U-shaped curve with PNI growth in patients aged over 60 years or complicated with COPD. On the one hand, both old age and COPD were proven to be independent risk factors for delirium. Aged people or those with COPD were more vulnerable to malnutrition [50, 51]. Thus, the front part of the U-shaped curve decreased due to the improvement in nutritional status, showing a reduced risk of delirium. On the other hand, PNI is not only an indicator representing malnutrition but also indicates immune status, which is also a risk factor for delirium [52]. Due to the malnourishment status caused by old age or COPD, serum albumin levels in these patients do not tend to be very high. Hence, the high PNI is more likely to be caused by the lymphocyte elevation, which indicates a stronger inflammatory response. So the rising part of the curve emerged. However, this opinion needs to be further studied and tested.

In conclusion, this study is in good agreement with previous research on the relationship between nutritional condition and the appearance of delirium. Despite its preliminary nature, this paper further verified the adverse effect of malnutrition on critically ill patients in the ICU. Furthermore, PNI as a tool to assess the probability of delirium in ICU patients has its advantages. First, PNI is easy to acquire due to the albumin and lymphocyte count were routine blood biochemistry examinations in on ICU admission, and it needs no more additional cost. Second, as an easily acquired index, PNI provide a now manner to alarm the occurence the delirium.Third, it may serve as a predictive indicator. Once the PNI is at a risky grade, additional measures can be applied to prevent the occurrence of delirium, to better protect these patients.

However, this study still has limitations. This paper is an observational study, which only allows to assess the association between PNI and delirium. Furthermore, heterogeneity may still exist due to the complex clinical condition of the ICU patient, although PSM was applied. To further explore the causal relationship between PNI and delirium, prospective cohort study should be designed and conducted in the future.

## 5. Conclusion

This article found that PNI is negatively associated with the prevalence of delirium in critically ill adults in the ICU. PNI is an independent risk factor for the incidence of delirium in adults in the ICU.

## Figures and Tables

**Table 1:** Basic clinical and laboratory characteristics of the study patients.

	G1	G2	G3	G4	*p* value
*N*	773	779	771	782	
Delirium	192 (24.8%)	223 (28.6%)	295 (38.3%)	366 (46.8%)	<0.001
Demographics and characteristics					
Age, years, median (IQR)	63.20 (49.53, 74.61)	68.25 (55.20, 79.04)	66.25 (55.20, 78.51)	65.07 (54.46, 75.23)	<0.001
Gender, male (%)	473 (61.2%)	434 (55.7%)	426 (55.3%)	447 (57.2%)	0.075
DM, yes (%)	233 (30.1%)	230 (29.5%)	217 (28.1%)	204 (26.1%)	0.290
COPD, yes (%)	65 (8.4%)	105 (13.5%)	110 (14.3%)	93 (11.9%)	0.002
CKD, yes (%)	109 (14.1%)	189 (24.3%)	185 (24.0%)	154 (19.7%)	<0.001
Basic vital signs					
Heart rate/min, median (IQR)	80.42 (70.13, 91.87)	82.80 (73.38, 96.29)	86.45 (76.00, 99.80)	92.05 (79.91, 103.67)	<0.001
Respiratory rate/min, median (IQR)	18.48 (16.54, 20.84)	19.34 (17.33, 21.88)	19.87 (17.38, 22.73)	20.48 (17.80, 23.81)	<0.001
SBP, mmHg, median (IQR)	121.31 (109.65, 133.40)	117.47 (107.52, 130.45)	114.54 (105.31, 126.50)	108.23 (101.26, 117.07)	<0.001
DBP, mmHg, median (IQRact)	69.42 (61.31, 78.54)	66.00 (58.96, 74.65)	64.21 (57.38, 71.47)	61.46 (55.57, 67.29)	<0.001
Temperature, °C, median (IQR)	36.85 (36.69, 37.04)	36.86 (36.69, 37.11)	36.89 (36.69, 37.19)	36.89 (36.67, 37.25)	0.004
SpO_2_, median (IQR)	96.91 (95.56, 97.93)	96.64 (95.23, 98.04)	96.67 (95.21, 98.05)	96.70 (95.36, 98.11)	0.350
Laboratory findings					
Glucose, mEq/L, median (IQR)	6.83 (5.85, 8.52)	7.07 (6.01, 8.81)	7.11 (5.89, 9.15)	6.94 (5.78, 9.31)	0.150
Hemoglobin, g/dL, median (IQR)	11.50 (9.35, 13.10)	10.50 (8.50, 11.90)	9.50 (7.90, 11.00)	8.20 (7.10, 9.90)	<0.001
Creatinine, mEq/L, median (IQR)	1.00 (0.80, 1.30)	1.10 (0.80, 1.70)	1.20 (0.80, 2.00)	1.30 (0.80, 2.30)	<0.001
Bicarbonate, mEq/L, median (IQR)	22.00 (19.00, 24.00)	21.00 (18.00, 23.00)	20.00 (17.00, 23.00)	19.00 (16.00, 22.00)	<0.001
Calcium, mg/dL, median (IQR)	8.60 (8.10, 9.00)	8.30 (7.80, 8.70)	8.00 (7.60, 8.40)	7.50 (7.00, 8.00)	<0.001
Chloride, mEq/L, median (IQR)	101.00 (98.00, 104.00)	101.00 (97.00, 104.00)	101.00 (96.00, 104.00)	100.00 (96.00, 105.00)	0.460
Potassium, mEq/L, median (IQR)	3.90 (3.60, 4.10)	3.90 (3.60, 4.30)	3.90 (3.50, 4.30)	3.80 (3.50, 4.30)	0.078
PT, s, median (IQR)	12.90 (11.70, 15.00)	13.75 (12.20, 16.80)	14.80 (12.90, 18.40)	16.10 (13.90, 21.10)	<0.001
PTT, s, median (IQR)	30.70 (28.10, 42.40)	31.70 (27.80, 44.30)	32.00 (28.20, 41.90)	33.75 (29.20, 44.20)	<0.001
ALT, IU/L, median (IQR)	23.00 (15.00, 43.00)	24.00 (15.00, 53.00)	29.00 (16.00, 67.00)	37.00 (18.00, 101.50)	<0.001
AST, IU/L, median (IQR)	29.00 (20.00, 56.00)	36.00 (22.00, 85.00)	44.00 (24.00, 107.00)	58.00 (29.00, 171.00)	<0.001
Bilirubin, mg/Dl, median (IQR)	0.60 (0.40, 0.90)	0.70 (0.40, 1.20)	0.70 (0.40, 1.60)	1.10 (0.50, 2.70)	<0.001

DM: diabetes mellitus; EHP: essential hypertension; CKD: chronic kidney disease; COPD: chronic obstructive pulmonary disease; SBP: systolic blood pressure; DBP: diastolic blood pressure; SpO_2_: saturation of the pulse oxygen; PT: prothrombin time; PTT: partial thromboplastin time; ALT: alanine aminotransferase; AST: aspartate aminotransferase.

**Table 2:** Odds ratio (95% CI) for delirium in different models.

	G1	G2	G3	G4	*P* for trend
Model 1	1.00 (reference)	1.21 (0.97, 1.52)	1.88 (1.51, 2.33)^*∗∗∗*^	2.66 (2.15, 3.30)^*∗∗∗*^	<0.001
Model 2	1.00 (reference)	1.14 (0.90, 1.45)	1.69 (1.33, 2.13)^*∗∗∗*^	2.26 (1.78, 2.87)^*∗∗∗*^	<0.001
Model 3	1.00 (reference)	1.06 (0.82, 1.38)	1.55 (1.19, 2.01)^*∗∗∗*^	1.94 (1.45, 2.59)^*∗∗∗*^	<0.001
Model 4	1.00 (reference)	1.04 (0.80, 1.36)	1.53 (1.17, 1.99)^*∗∗*^	1.93 (1.44, 2.59)^*∗∗∗*^	<0.001

Model 1: univariate analysis; model 2: adjusted for heart rate, SBP, DBP, temperature, and SpO_2_; model 3: further adjusted for age, grade, gender, blood glucose, hemoglobin, creatinine, bicarbonate, calcium, chloride, potassium, PT, PTT, AST, ALT, and total bilirubin; model 4: further adjusted for DM, COPD, CKD. ^*∗*^*P* < 0.05, ^*∗∗*^*P* < 0.01, ^*∗∗∗*^*P* < 0.001.

**Table 3:** Subgroup analysis of association between PNI and delirium.

	G1	G2	G3	G4	*p* values for interactions
Gender					>0.05
Female	1.00 (reference)	1.03 (0.67, 1.57)	1.25 (0.81, 1.92)	1.85 (1.14, 3.01)^*∗*^	
Male	1.00 (reference)	1.07 (0.75, 1.52)	1.80 (1.27, 2.55)^*∗∗∗*^	2.13 (1.46, 3.12)^*∗∗∗*^	
DM					>0.05
No	1.00 (reference)	1.12 (0.81, 1.54)	1.53 (1.11, 2.12)^*∗*^	2.01 (1.41, 2.88)^*∗∗∗*^	
Yes	1.00 (reference)	0.82 (0.50, 1.36)	1.53 (0.94, 2.48)	1.68 (0.98, 2.90)	
COPD					>0.05
No	1.00 (reference)	1.11 (0.83, 1.48)	1.69 (1.27, 2.26)^*∗∗∗*^	1.98 (1.44, 2.73)^*∗∗∗*^	
Yes	1.00 (reference)	0.62 (0.28, 1.39)	0.86 (0.35, 2.10)	1.94 (0.59, 6.45)	
CKD					>0.05
No	1.00 (reference)	1.05 (0.78, 1.42)	1.61 (1.19, 2.18)^*∗∗*^	1.93 (1.39, 2.69)^*∗∗∗*^	
Yes	1.00 (reference)	1.04 (0.55, 1.96)	1.34 (0.72, 2.50)	1.97 (0.98, 3.94)	
Age grade					>0.05
<45	1.00 (reference)	1.70 (0.79, 3.70)	2.07 (0.91, 4.71)	1.37 (0.55, 3.43)	
<60	1.00 (reference)	1.70 (0.79, 3.70)	2.07 (0.91, 4.71)^*∗∗*^	1.37 (0.55, 3.43)^*∗∗*^	
≥60	1.00 (reference)	1.70 (0.79, 3.70)	2.07 (0.91, 4.71)	1.37 (0.55, 3.43)^*∗∗∗*^	

DM: diabetes mellitus; EHP: essential hypertension; CKD: chronic kidney disease; COPD: chronic obstructive pulmonary disease; ^*∗*^*P* < 0.05, ^*∗∗*^*P* < 0.01, ^*∗∗∗*^*P* < 0.001.

## Data Availability

The datasets used for the analysis in the current study are available from the corresponding author upon a reasonable request. And it can also be accessed at the website: https://mimic.mit.edu/.

## Supplementary Materials

Supplementary Materials Supplementary figure. Standardized bias (%) across covariates; all standardized biases after propensity score matching were less than 0.1. Supplementary table. Basic clinical and laboratory characteristics after propensity score matching. DM: diabetes mellitus; EHP: essential hypertension; CKD: chronic kidney disease; COPD: chronic obstructive pulmonary disease; SBP: systolic blood pressure; DBP: diastolic blood pressure; SpO_2_: saturation of the pulse oxygen; PT: prothrombin time; PTT: partial thromboplastin time; ALT: alanine aminotransferase; AST: aspartate aminotransferase.
