# Supplementary material for: Clinical Significance of the Prognostic Nutritional Index in Predicting Delirium among Critically Ill Patients: A Retrospective Cohort Study
Source: Crit Care Res Pract. 2024 May 11;2024:3807532. doi: 10.1155/2024/3807532 (PMC11102111; doi:10.1155/2024/3807532)
Supplement: Supplementary Materials — Supplementary figure. Standardized bias (%) across covariates; all standardized biases after propensity score matching were less than 0.1. Supplementary table. Basic clinical and laboratory characteristics after propensity score matching. DM: diabetes mellitus; EHP: essential hypertension; CKD: chronic kidney disease; COPD: chronic obstructive pulmonary disease; SBP: systolic blood pressure; DBP: diastolic blood pressure; SpO2: saturation of the pulse oxygen; PT: prothrombin time; PTT: partial thromboplastin time; ALT: alanine aminotransferase; AST: aspartate aminotransferase. [file 3807532.f1.zip › Supplemental Table Basic clinical and laboratory characteristics after PSM.docx]

**Supplemental Table Basic clinical and laboratory characteristics after PSM**

|  | **PNI≥37.32** | **PNI<37.32** | **p** |
| --- | --- | --- | --- |
| **N** | 730 | 730 |  |
| **Delirium** | 228 (31.2%) | 289 (39.6%) | <0.001 |
| **Demographics and characteristics** |  |  |  |
| Age, years, median (IQR) | 66.92 (54.86, 77.69) | 66.38 (56.21, 77.38) | 0.820 |
| Gender, male, (%) | 403 (55.2%) | 410 (56.2%) | 0.710 |
| DM, yes, (%) | 225 (30.8%) | 218 (29.9%) | 0.690 |
| COPD, yes, (%) | 109 (14.9%) | 97 (13.3%) | 0.370 |
| CKD, yes, (%) | 174 (23.8%) | 168 (23.0%) | 0.710 |
| **Basic vital signs** |  |  |  |
| Heart rate, /min, median (IQR) | 86.91 (76.52, 98.65) | 85.54 (75.28, 99.43) | 0.380 |
| Respiratory rate, /min, median (IQR) | 19.69 (17.59, 22.54) | 19.67 (17.31, 22.43) | 0.560 |
| SBP, mmHg, median (IQR) | 113.63 (105.00, 124.54) | 112.68 (104.66, 125.81) | 0.830 |
| DBP, mmHg, median (IQR) | 63.65 (57.29, 70.96) | 63.82 (57.75, 70.88) | 0.740 |
| Temperature, °C, median (IQR) | 36.89 (36.69, 37.13) | 36.86 (36.66, 37.16) | 0.230 |
| SpO_2_, median (IQR) | 96.74 (95.28, 98.06) | 96.72 (95.28, 98.04) | 0.910 |
| **Laboratory findings** |  |  |  |
| Glucose, mEq/L, median (IQR) | 7.15 (6.03, 8.85) | 6.98 (5.79, 9.11) | 0.260 |
| Hemoglobin, g/dL, median (IQR) | 9.30 (7.70, 10.90) | 9.50 (8.10, 11.00) | 0.086 |
| Creatinine, mEq/L, median (IQR) | 1.20 (0.90, 1.90) | 1.20 (0.80, 1.90) | 0.210 |
| Bicarbonate, mEq/L, median (IQR) | 20.00 (17.00, 23.00) | 20.00 (17.00, 23.00) | 0.850 |
| Calcium, mg/dL, median (IQR) | 8.10 (7.60, 8.50) | 8.00 (7.60, 8.40) | 0.150 |
| Chloride, mEq/L, median (IQR) | 101.00 (97.00, 104.00) | 101.00 (97.00, 104.00) | 0.640 |
| Potassium, mEq/L, median (IQR) | 3.90 (3.50, 4.30) | 3.80 (3.50, 4.30) | 0.430 |
| PT, s, median (IQR) | 14.60 (12.70, 18.30) | 14.90 (13.10, 18.30) | 0.140 |
| PTT, s, median (IQR) | 31.90 (28.00, 43.60) | 32.50 (28.50, 43.90) | 0.200 |
| ALT, IU/L, median (IQR) | 25.00 (15.00, 56.00) | 31.00 (17.00, 72.00) | 0.033 |
| AST, IU/L, median (IQR) | 39.00 (23.00, 98.00) | 46.00 (26.00, 108.00) | 0.023 |
| Bilirubin, mg/Dl, median (IQR) | 0.70 (0.40, 1.30) | 0.75 (0.40, 1.90) | 0.018 |

DM: diabetes mellitus; EHP: essential hypertension; CKD: chronic kidney disease; COPD：chronic obstructive pulmonary disease; SBP: systolic blood pressure; DBP: diastolic blood pressure; SpO2: saturation of the pulse oxygen; PT: prothrombin time; PTT: partial thromboplastin time; ALT: alanine aminotransferase; AST: aspartate aminotransferase.
