# Supplementary figures and images for: Clinical Significance of the Prognostic Nutritional Index in Predicting Delirium among Critically Ill Patients: A Retrospective Cohort Study
Source: Crit Care Res Pract. 2024 May 11;2024:3807532. doi: 10.1155/2024/3807532 (PMC11102111; doi:10.1155/2024/3807532)

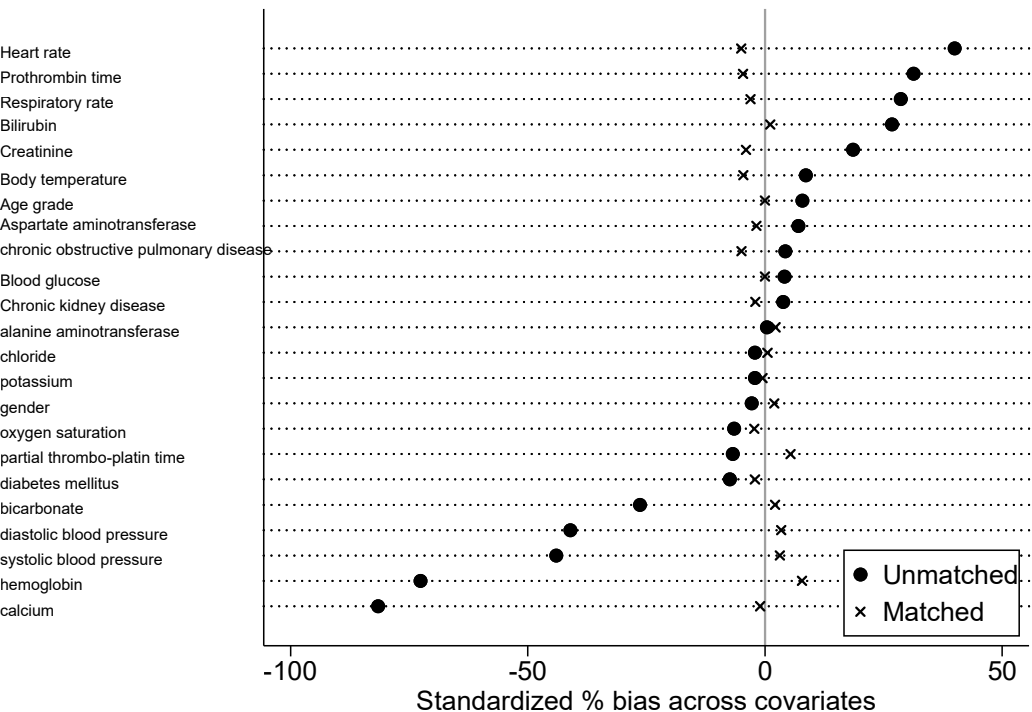

Supplement: Supplementary Materials — Supplementary figure. Standardized bias (%) across covariates; all standardized biases after propensity score matching were less than 0.1. Supplementary table. Basic clinical and laboratory characteristics after propensity score matching. DM: diabetes mellitus; EHP: essential hypertension; CKD: chronic kidney disease; COPD: chronic obstructive pulmonary disease; SBP: systolic blood pressure; DBP: diastolic blood pressure; SpO2: saturation of the pulse oxygen; PT: prothrombin time; PTT: partial thromboplastin time; ALT: alanine aminotransferase; AST: aspartate aminotransferase. [file 3807532.f1.zip › supplemental fig.pdf]
